# Whitmore's Disease: A Case Report of Melioidosis Triggering Hemophagocytic Lymphohistiocytosis

**DOI:** 10.7759/cureus.65948

**Published:** 2024-08-01

**Authors:** Kalyan Bojanapati, Rucha Gohil, Chakradhar Siddem, J Kumar

**Affiliations:** 1 Internal Medicine, SRM Medical College Hospital and Research Centre (SRMIST), Chengalpattu, IND; 2 General Medicine, SRM Medical College Hospital and Research Centre (SRMIST), Chengalpattu, IND

**Keywords:** burkholderia pseudomallei, anemia, melioidosis, splenic abscess, pneumonia, fever, hemophagocytic lymphohistiocytosis (hlh)

## Abstract

Melioidosis is a rare disease caused by *Burkholderia pseudomallei*, which has recently acquired prominence in India as an emerging pathogen. It is a gram-negative bacteria found in soil. As delayed diagnosis and treatment are linked to increased mortality, early diagnosis is crucial. We present here a unique instance of melioidosis that was made worse by a rare disorder known as hemophagocytic lymphohistiocytosis (HLH), which necessitates early treatment.

## Introduction

*Burkholderia pseudomallei*, the causative agent of melioidosis, is an uncommon infection prevalent throughout Southeast and South Asia, including India. The majority of cases have been documented in Thailand and Singapore [1].

This is a disease that is transmitted primarily through percutaneous inoculation of contaminated water. In endemic areas, ingestion of contaminated water is also an important route of transmission of this hazardous infection [2]. Though most of the cases are asymptomatic, severe forms of illness due to melioidosis are more often in patients with diabetes, chronic kidney/liver disease, and in patients who abuse alcohol [3]. This disease can have an acute presentation or a chronic presentation, with pneumonia being the common manifestation. Before starting empirical anti-tuberculosis therapy for chronic pneumonia, it is crucial to rule out other possible causes, such as melioidosis, especially in patients with a history of diabetes or chronic liver or renal disease in endemic regions like India. Pulmonary tuberculosis is one of the important differential diagnoses in cases of chronic presentation.

Hemophagocytic lymphohistiocytosis (HLH) is primarily a pediatric syndrome affecting one in 3,000 children admitted to a tertiary care pediatric hospital. Though rare, HLH is also reported in adult patients of all age groups and is a dreadful and life-threatening complication that occurs due to excessive immune activation, which can be familial or sporadic and can be triggered by a variety of events that disrupt the immune homeostasis and is commonly triggered by viral infections, but here we report a case of melioidosis, which is a bacterial infection that is complicated with HLH.

## Case presentation

**Figure 1:**
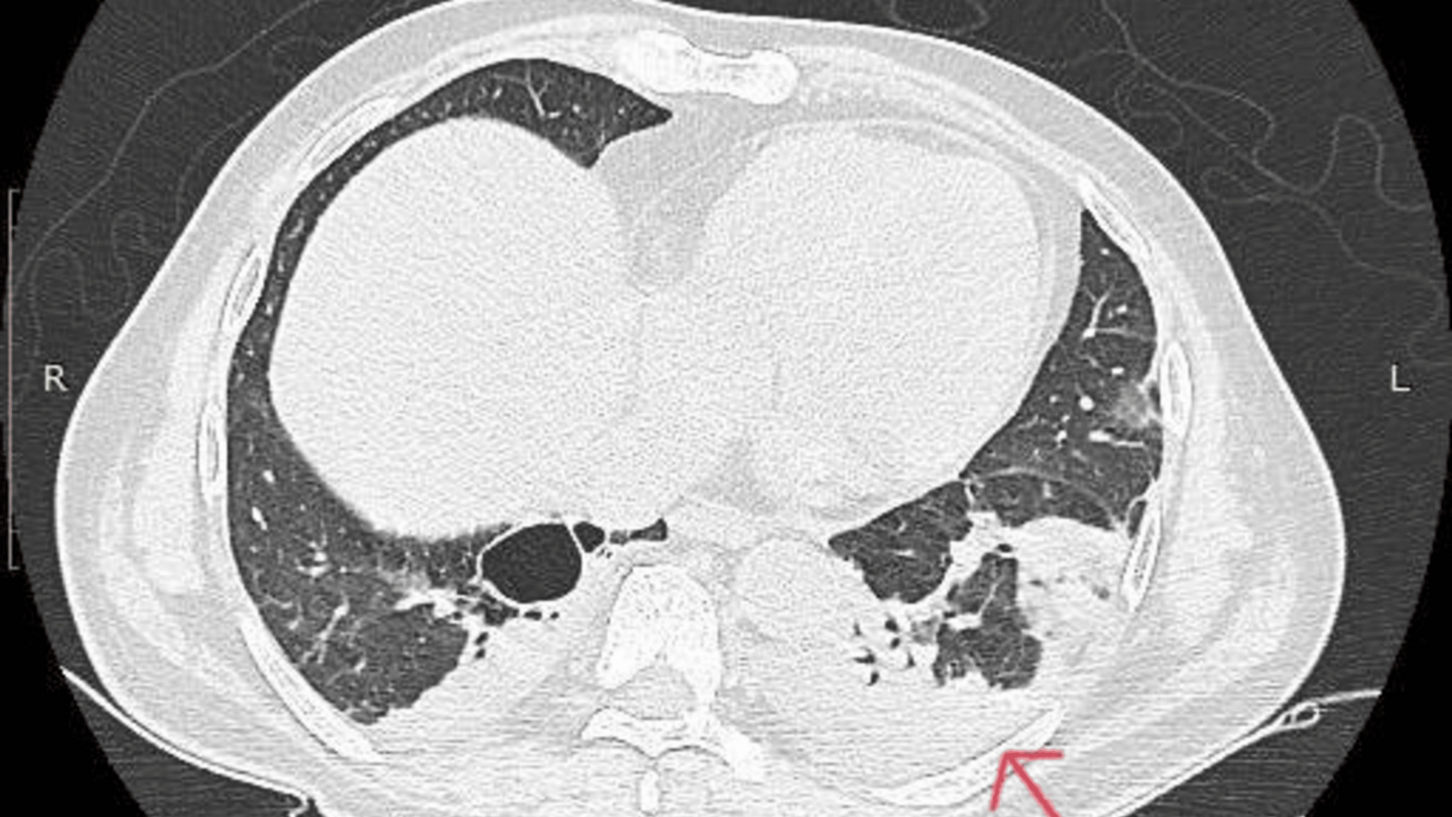
A CT scan of the chest shows multiple lung nodules with patchy areas of consolidation and ground glass opacities, as illustrated above by the red arrow.

A 60-year-old male patient who was diabetic and an alcoholic hailing from the Chengalpattu district of the state of Tamil Nadu, India, presented to the emergency department with complaints of fever for 10 days associated with loose stools for three days and dry cough for two days with a blood pressure of 90/60 mmHg and a heart rate of 106 beats/min on arrival. On assessment, the quick SOFA (qSOFA) score was found to be two, and the patient was initially diagnosed with sepsis with unknown foci, for which the patient was given ceftriaxone empirically as it is a broad-spectrum antibiotic, and blood cultures were sent. Further, the patient developed desaturation with the need for oxygen support on day two, but the chest X-ray and respiratory examination were unremarkable at that point, and blood gas analysis revealed hypoxemia with partial pressure of oxygen (PaO_2_) of 72 mmHg. The initial culture was reported with coagulase-negative staphylococcus (CoNS), which was sensitive to clindamycin, following which the patient was started on the same on day six, despite which the patient deteriorated with increased oxygen requirement on day 10. Respiratory examination on day 10 revealed new crepitations for which a CT scan of the chest was done, which revealed changes suggestive of pneumonia (Figure 1).

Because of pneumonia, the patient was started on piperacillin-tazobactam at the dose of 4.5 gm every six hours with added azithromycin, despite which there was no improvement in the patient's clinical status and the patient went on needing noninvasive ventilation support. Additionally, the patient also started developing cellulitis of the right lower limb.

**Figure 2:**
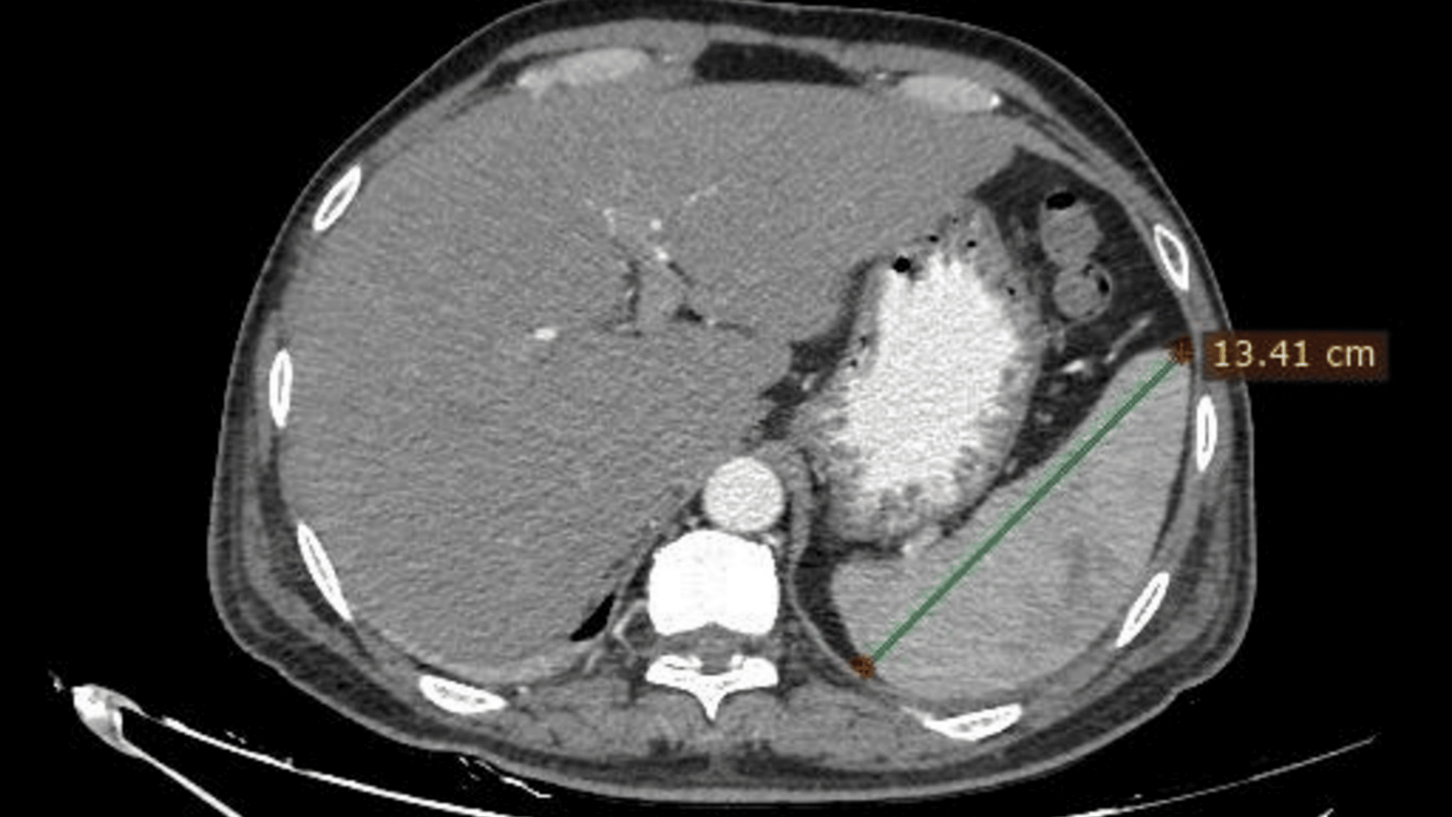
A CT scan of the abdomen shows splenomegaly, as represented by the green tracer above.

Further, the patient developed abdominal pain for which a CT scan of the abdomen was done, which revealed splenomegaly (Figure 2) with a splenic abscess. Further, given the splenic abscess, echocardiography was done to rule out infective endocarditis, which revealed no abnormality, and the abscess was drained with the help of ultrasound-guided percutaneous drainage. The pus was sent for culture and sensitivity testing along with the blood cultures from three different sites in the suspicion of melioidosis due to the background pneumonia, splenic abscess, splenomegaly, and cellulitis.

**Figure 3:**
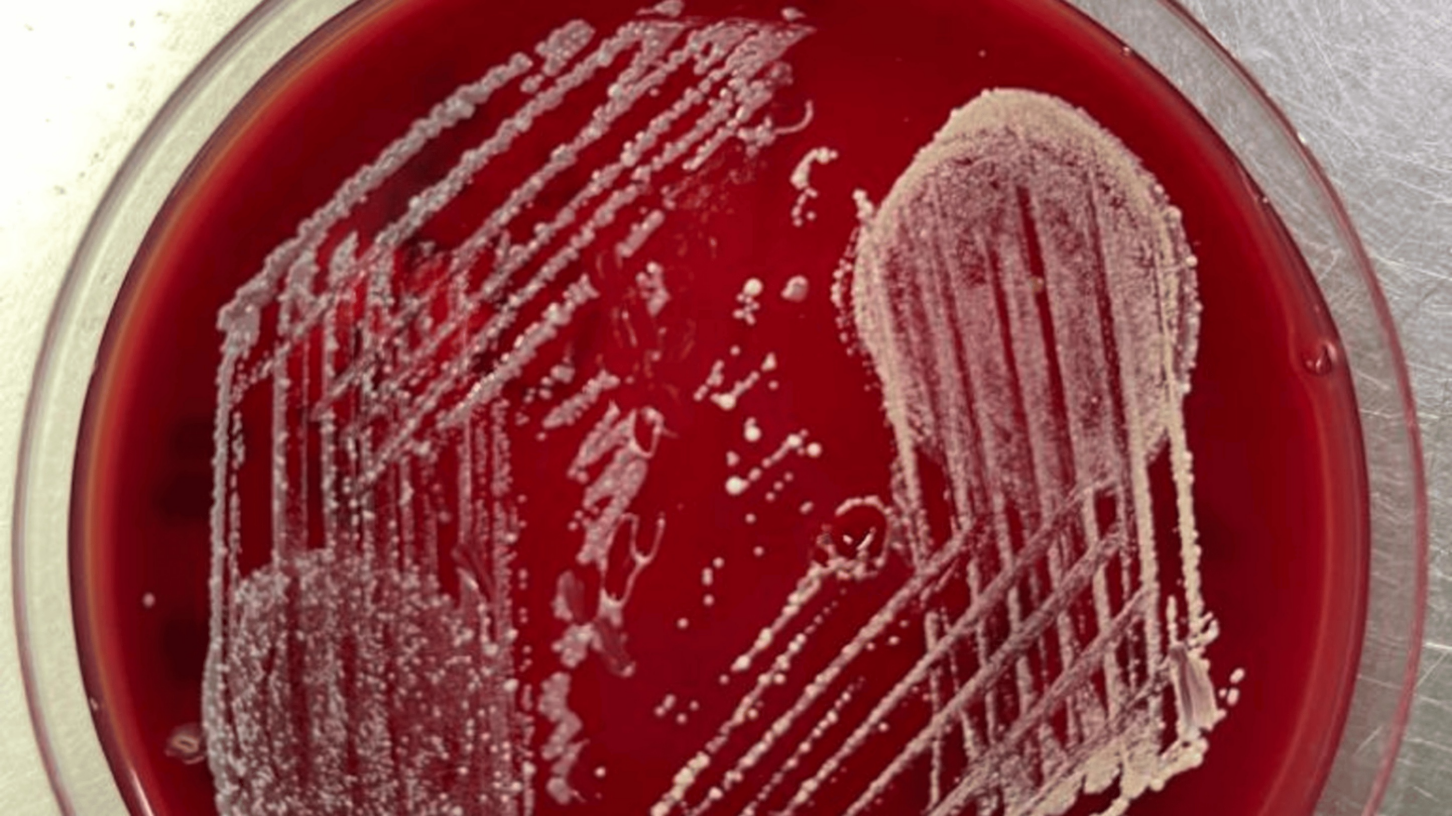
Colonies of Burkholderia psuedomallei organism on MacConkey agar

Subsequently, *Burkholderia pseudomallei *(Figure 3) was discovered in the blood and pus cultures; this organism was sensitive to ceftazidime, so the patient was started on it at the dose of 2 gm every six hours along with trimethoprim-sulfamethoxazole at the dose of 240 mg every 12 hours. However, the patient continued to deteriorate despite treatment for five days with the above-mentioned antibiotics, developing bicytopenia with a drop in hemoglobin and platelets and a fever that did not resolve despite receiving the appropriate treatment.

In the suspicion of HLH, samples for ferritin and triglycerides were sent, and the results showed elevated levels of 3,360 ng/ml and 301 mg/dl, respectively (Table 1). 

**Table 1:** Blood parameters on serial monitoring Hb: hemoglobin; TGL: triglycerides

Investigations	Day 1	Day 7	Day 19	Reference values
Hb	12 g/dL	11.6 g/dL	7.5 g/dL	13-17 g/dL
Platelet count	1,80,000/cu.mm	1,60,000/cu.mm	64,000/cu.mm	1,50,000-4,10,000/cu.mm
Ferritin			3360 ng/mL	24-336 ng/mL
TGL			301 mg/dL	50-150 mg/dL

The bone marrow examination findings were also supportive of the diagnosis of HLH. This was based on the presence of evidence of hemophagocytosis in bone marrow with elevated triglycerides and ferritin in the background of splenomegaly and bicytopenia. Consequently, aggressive treatment was started for HLH with dexamethasone at a dose of and etoposide at a dose of 200 mg twice weekly for two weeks followed by 200 mg weekly for six weeks (100-150 mg/sqm), following which the patient improved with subsidence of fever in five days followed by improvement in cytopenia and respiratory functions with no further requirement of ventilatory support.

## Discussion

Melioidosis is a rare disease that is associated with significant mortality and morbidity if not diagnosed and treated early. Even though most of the cases are asymptomatic, some are symptomatic, among which most patients have acute presentation, with pneumonia being the most common manifestation with high-grade fever and respiratory distress with or without circulatory failure with low blood pressure [4].

Melioidosis can also have a subacute to chronic presentation with cough, hemoptysis, and imaging findings similar to pulmonary tuberculosis, so before starting an empirical antitubercular therapy, it is always important to rule out this condition, particularly in patients with risk factors such as diabetes and chronic renal/liver disease. A cutaneous form of melioidosis is also seen in some cases, which manifests as pustules and macular lesions [5].

Cellulitis secondary to melioidosis is also reported, particularly in the Asian subcontinent, though it’s not that common [6]. Other rare manifestations include neurological insult with encephalomyelitis, meningitis, myelitis, cerebral abscess, and abscess within the internal organs such as the liver and spleen [7]. In rare cases, there can also be musculoskeletal involvement in melioidosis with septic arthritis and osteomyelitis. While coming to the diagnosis, it is important, particularly in endemic regions such as India, to have a clinical suspicion of this rare and dreadful infection in the background of acute or chronic pneumonia with splenic abscess and other skin manifestations. The samples can be taken from blood, sputum, or pus/fluid from drainable abscesses for cultures. The treatment includes an intensive phase and an eradication phase. The duration of antibiotics varies depending upon the site of involvement and severity of the disease, with ceftazidime and trimethoprim/sulfamethoxazole as the treatment of choice and meropenem/imipenem as alternatives for the patient who tends to have persistent fever despite a week of ceftazidime.

Hemophagocytic lymphohistiocytosis is a life-threatening complication that is associated with excessive immune activation and is common in children but can be seen in all age groups. Viral infections are the most common triggers for HLH, such as cytomegalovirus, parvovirus, and herpes simplex virus [8,9]. Here we report a rare bacterial infection triggering this dreadful complication. Another rare presentation has been reported in the American Journal of Tropical Medicine [10].

Hemophagocytic lymphohistiocytosis can be familial or sporadic and is typically associated with anemia and thrombocytopenia with median hemoglobin of 7.2 g/dl and median platelets of 69,000/microL [11]. Hemophagocytic lymphohistiocytosis was suspected due to the development of anemia (hemoglobin of 7.5 g/dl) and thrombocytopenia (platelets of 64,000/microL). Serum ferritin and triglycerides were sent, along with which a bone marrow biopsy was performed. The results were consistent with HLH in our case, as our case met six of nine criteria, inclusive of fever greater than 38.5°C, splenomegaly, peripheral blood cytopenia, hypertriglyceridemia, hemophagocytosis in bone marrow, and high ferritin (more than 500 ng/mL). Other criteria that are not present in our case include low NK cell activity, elevated CXCL9, and elevated soluble CD25. If the condition does not improve after treating the underlying trigger, we need to aggressively treat it with immunosuppressants like steroids, preferably dexamethasone and etoposide.

## Conclusions

This case focuses on the need for clinical suspicion of a rare bacterial infection caused by *Burkholderia pseudomallei*, as early diagnosis and treatment are important to reduce the mortality and morbidity associated with this condition, particularly in endemic areas such as India, especially Tamil Nadu, in a patient with pneumonia in the background of risk factors such as diabetes, alcoholism, and renal/liver failure. It is also important to know that it can even trigger a rare and dreadful complication called HLH, which calls for an early diagnosis and aggressive treatment, as delayed initiation of treatment is associated with significant mortality. By this, we present the diagnostic challenges in melioidosis, and it is also important to recognize that complications such as hemophagocytic lymphohistiocytosis can be triggered by bacterial infections such as melioidosis apart from viral infections.
